# A New Definition of Thrombocytopenia Following Transcatheter Aortic Valve Implantation: Incidence, Outcome, and Predictors

**DOI:** 10.3390/jcdd9110388

**Published:** 2022-11-09

**Authors:** Francesco Pollari, Stine Horna, Magnus Rottmann, Christian Langhammer, Thomas Bertsch, Theodor Fischlein

**Affiliations:** 1 Cardiac Surgery, Cardiovascular Department, Klinikum Nürnberg—Paracelsus Medical University, 90471 Nuremberg, Germany; 2 Institute of Clinical Chemistry, Laboratory Medicine and Transfusion Medicine, Klinikum Nürnberg—Paracelsus Medical University, 90471 Nuremberg, Germany

**Keywords:** TAVI, TAVR, thrombocytopenia, blood disorders, aortic valve disease

## Abstract

Background: The aim of this study was to assess the incidence, outcomes, and risk factors associated with thrombocytopenia following TAVI according to a corrected platelet count (CPC), to avoid the bias of hemodilution/concentration. Methods: We analyzed patients who underwent TAVI in our center between 2009 and 2018. The study population were divided into three groups: none (NT), mild (MT), and severe (ST) postoperative thrombocytopenia. Primary outcomes were bleedings, length of hospital stay, and mortality. A multivariate logistic regression was performed to assess risk factors for ST. Results: A total of 907 patients were included in the analysis. MT was observed in 28.1% and ST in 2.6% of all patients. The following clinical outcomes were recorded: incidence of life-threatening and major bleeding (NT = 14.2%, MT = 20.8%, ST = 58.3%), the median length of hospital stay (NT = 8, MT = 10, ST = 14 days), in-hospital mortality (NT = 3.9%, MT = 6.3%, ST = 16.7%), and the overall significance in comparison with NT (*p* < 0.05). The logistic regression showed ST was associated with preoperative CPC, transapical access, diabetes mellitus, and the critical preoperative state. Conclusions: Worse clinical outcomes are associated with both MT and ST after TAVI. In particular, ST is associated with higher in-hospital and 30-day mortality. Management of modifiable baseline and procedural variables may improve this outcome.

## 1. Introduction

The treatment for severe aortic valve stenosis has markedly changed in the last decade. Transcatheter aortic valve implantation (TAVI) emerged as the primary option over surgical aortic valve replacement in many categories of patients. The comprehension of postprocedural complications and their effect on the outcomes is particularly important, as indications are currently expanding to a younger population.

Thrombocytopenia following TAVI is not uncommon. Dvir and colleagues reported a significant decrease in platelet count under 100,000/μL after TAVI in one-third of the patients [1]. Jilaihawi and colleagues also reported major thrombocytopenia in over one-third of the study population [2]. However, evidence regarding its incidence, consequences, and possible risk factors is scant; limited to small samples; or biased by periprocedural fluid management causing hemodilution [3].

We aimed to assess the incidence, outcomes, and predictors of in-hospital thrombocytopenia following TAVI in a large single-center study.

## 2. Materials and Methods

**Figure 1:**
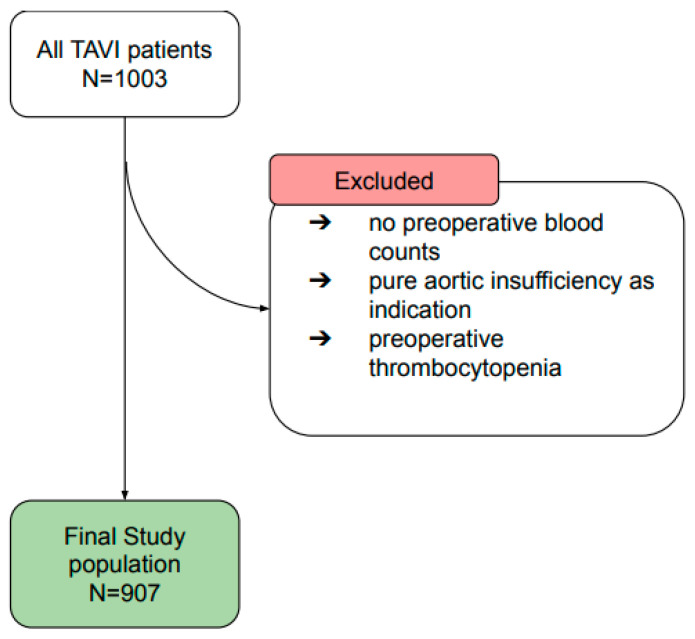
A flowchart showing the selection process of the study population.

The study population comprised 1003 patients, who underwent TAVI in the Heart and Vascular Center of Nuremberg general hospital between July 2009 and April 2018. A total of 62 patients, whose blood values could no longer be traced before the TAVI, were excluded, as well as 1 patient with pure aortic regurgitation, and 20 patients who preoperatively had chronic thrombocytopenia (<100,000/μL) (Figure 1).

In total, the data from 907 patients were retrospectively evaluated. All procedures were conducted in a hybrid operating room under fluoroscopic control (Artis Zeego System, Siemens AG, Erlangen, Germany), general anesthesia, periprocedural TEE, and a cardiac perfusionist with a ready-to-use cardiopulmonary bypass on site. All implantations were performed by a team composed of an interventional cardiologist and a cardiac surgeon. Patients received either a balloon-expanding (Sapien, SapienXT, Sapien3, Edwards Lifesciences Inc., Irvine, CA, USA) or a self-expanding valve prosthesis (e.g., Medtronic CoreValve, CoreValve Evolut R, CoreValve EvolutPro, Engager, Medtronic, Minneapolis, MN: Acurate, Acurate neo, Symetis SA, Ecublens, Switzerland). The selection of the prosthesis was individually made for each patient by the heart team, based on echocardiography and CT findings. Per institutional protocol, the transfemoral approach (TF-TAVI) was the preferred standard access. In case of severe peripheral arteriopathy, transapical access was performed (TA-TAVI). After the procedure, all patients were loaded with 300 mg clopidogrel, and starting from day 1, received a double antiaggregation therapy consisting of 100 mg/die acetylsalicylic acid, and 75 mg/die clopidogrel for three months. Moreover, low-molecular-weight heparin was applied during the hospital stay, as prophylaxis for deep vein thrombosis. All patients provided written informed consent for the anonymous use of their data, and the study was approved by our institutional study center (SZ_D_125.20-I-6). The study protocol conforms to the ethical guidelines in the Declaration of Helsinki.

### 2.1. Platelet Counts and Thrombocytopenia

The platelet count was routinely measured at admission before the index procedure, on the day of the procedure (day “0”), and on postoperative days 1–5. In addition, the platelet count was measured one last time on the day of discharge. In order to better compare the postoperative values, the corrected platelet count (CPC; according to the formula = Platelet count t × (Hematocrit t/Hematocrit preoperatively), where t corresponds to a determinant postoperative day) was used [3]. In this way, we could reduce the risk of bias due to perioperative confounders (such as hemodilution or concentration), which often occur in the management of the postoperative phase, and could influence the platelet count. If there was more than one platelet count per day, the lowest of the two was selected for analysis. A CPC of less than 100,000/μL was defined as mild thrombocytopenia (MT). Severe thrombocytopenia (ST) was defined below 50,000/μL [4]. Based on the onset of postoperative thrombocytopenia, the study population was divided into three groups: NT (no thrombocytopenia), MT, and ST.

### 2.2. Outcomes

The following outcomes were recorded based on the Valve Academic Research Consortium-2 (VARC-2) recommendations [5]: length of hospital stay, life-threatening bleeding, major bleeding, in-hospital, and 30-days mortality.

The primary aim of the study was to assess the incidence of the above-mentioned outcomes, according to the grade of thrombocytopenia. The secondary aim was to assess risk factors for the onset of in-hospital severe thrombocytopenia.

### 2.3. Statistical Analysis

Data consistency was checked, and data were screened for outliers by using quantile plots. Continuous variables were tested for normality by using the Kolmogorov–Smirnov test. Cross-tabulation tables were computed and tested by using Pearson’s chi square, an M-L test, and a Wilcoxon–Mann–Whitney test, for two multinomial. Kruskal–Wallis ANOVA and Mann–Whitney U tests were applied. If test assumptions were not fully met, exact *p*-values based on Monte Carlo methods were used. Multivariate logistic regression analyses, and odds ratios (OR) with corresponding 95% confidence intervals (CI), were computed for predictors of severe thrombocytopenia. The proportional hazards (PHs) assumption was checked to meet all variables included in the model. To set up a multivariate model, only variables with univariate *p*-values < 0.1 were selected. All reported tests were two-sided, and *p*-values < 0.05 were considered statistically significant. All statistical analyses in this report were performed using SPSS software (IBM SPSS Statistics, Release 25, SPSS Inc., Chicago, IL, USA).

## 3. Results

Based on the noncorrected platelet count, out of the 907 patients evaluated, 109 (12%) patients developed mild thrombocytopenia and 5 (1%) developed severe thrombocytopenia. Table S1 shows the variation in noncorrected platelet count in the total study population and subgroups.

The use of CPC showed a postoperatively higher incidence of MT (255 patients, 28.11%) and ST (24 patients, 2.65%). Table 1 shows the baseline characteristics of the study population and of the subgroups according to the CPC. The mean age was 81.84 (±5.94), the BMI was 27.18 (±4.98), and 48.07% were women. The mean Euroscore II was 8.9% (±8.5). The majority of patients (847 patients, 93.38%) underwent an elective procedure. The mean hematocrit value and platelet count were 36.78%, and 224,460/µL, respectively. Except for a lower incidence of women in the MT group, the differences in the three study groups were mainly observed in laboratory and procedural parameters. Both the MT and ST patients showed higher preoperative hematocrit values and lower platelet count than NT patients.

**Figure 2:**
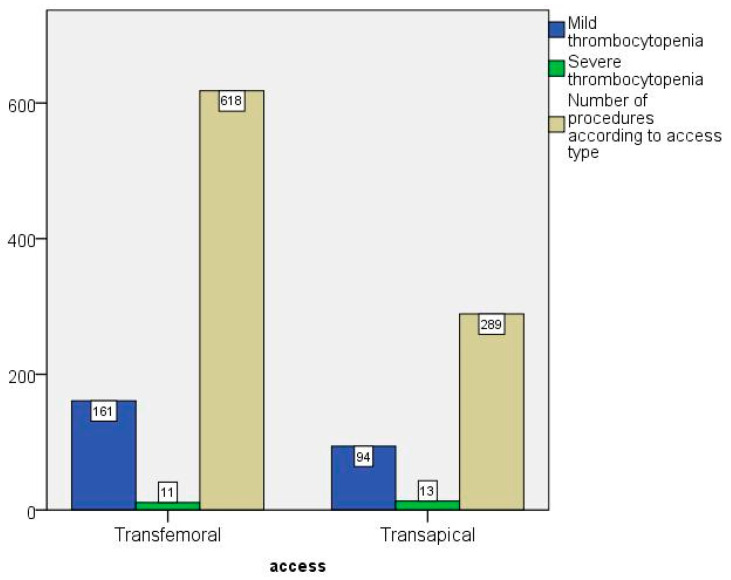
Incidence of mild (MT) and severe (ST) thrombocytopenia according to transfemoral (TF) or transapical (TA) access.

Most of the procedures (68.14%) were performed with the transfemoral approach (Figure 2).

**Figure 3:**
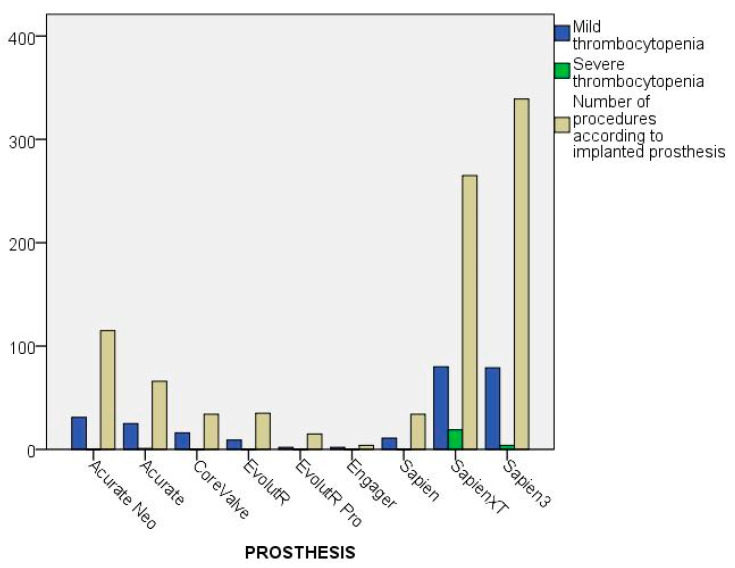
Boxplot showing the evolution of corrected platelet counts sorted by access and postoperative days.

Only 31.86% of all procedures were performed transapically. In the ST group, however, the proportion of TAVIs performed transapically was 54.17%. About 70% of all valve prostheses used were balloon-expanding valves. Figure 3 shows the outcome depending on the type of valve prosthesis. While MT could be found in all prosthesis groups (self-expandable = 31.6%; balloon-expandable = 26.6%), severe thrombocytopenia was mainly observed in the balloon-expandable group (3.6%). In comparison, only one person in the self-expandable group had ST (0.37%). A total of 53 patients (5.84%) received valve-in-valve implantation. In 91.07%, a valvuloplasty was performed before the new artificial heart valve was implanted. The proportions were also roughly the same in the different groups. In 271 patients (29.88%), at least one post-dilation had to be performed because of residual paravalvular leaks. 

**Figure 4:**
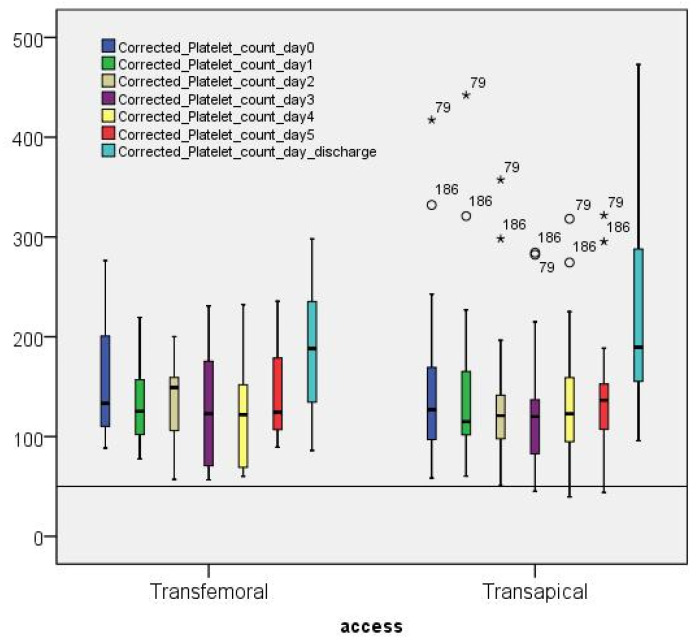
Bar chart showing the distribution of mild (MT) and severe (ST) thrombocytopenia according to implanted prosthesis. Asterisk and circles show outliners.

Figure 4 and Table S2 show the corrected platelet counts (CPCs) sorted by days and access. The detailed CPC values according to the study groups can be found in Table S3. Generally, lower CPC values were found in the group with transapical access compared with the transfemoral group. The thrombocyte nadir (the lowest platelet count) in both accesses (TF and TA) was observed on the third day postoperatively (134,960/μL ± 65.86).

The ST group showed a higher decrease in percent on the operative day “0” (≈54%; 82,150/μL ± 44,070), while the lowest mean value was observed on the fourth postoperative day (57,010/μL ± 14,130).

The nadirs of the NT group (163,800/μL ± 65,620) and the MT group (90,790/μL ± 25,510) were observed on the third postoperative day, which corresponded to 66.26% and 52.52% of the initial value of the platelets, respectively. Both the groups (NT and MT) regained their platelet counts on the day of discharge (245,800/μL ± 93,860, and 174,080/μL ± 73,430, respectively). However, the group with severe thrombocytopenia only gained 78% of the initial value (138,000/μL ± 94,800).

A total of 165 patients received red blood transfusions, 11 (45.83%) of them in the ST group. In the group with mild thrombocytopenia, the proportion was 21.18%, which was bigger than in the group without thrombocytopenia. Seven patients received FFPs. Twelve patients received platelet concentrates.

Table 2 shows the incidence of outcomes according to study group. Patients with MT and ST stayed significantly longer (3.73 days ± 6.18 and 4.75 days ± 5.46, respectively) in the intensive care unit than those in the NT group (2.33 days ± 3.8). Life-threatening bleeding occurred in 4.08% of the total population, and the proportion increased the more severe the thrombocytopenia became (mild thrombocytopenia 6.6%; severe thrombocytopenia 25%). A total of 15 patients suffered a clinically detectable stroke, but none of these occurred in the ST group. Life-threatening bleeding, in-hospital stay, and the 30-day mortality were significantly higher in the ST group than in the other study groups.

The multivariate logistic regression of the potential risk factors for the group with severe is shown in Table 3. The baseline platelet count, transapical access route, the noninsulin diabetes mellitus, and critical preoperative state (defined according to Euroscore definition) were found to be statistically associated with the onset of ST.

## 4. Discussion

The main findings of the present study are: (i) thrombocytopenia commonly occurs after TAVI; (ii) its onset adversely affects the prognosis of the patients; (iii) we found an association with baseline as well as procedural variables that could improve the prediction and avoidance of this complication.

Thrombocytopenia following interventions of the aortic valve is a well-recognized phenomenon in cardiac surgery, and its incidence was reported as being between 21% and 71% [6]. In general, a decrease in the corrected platelet count to a nadir on day 3 could be observed in all groups. Then, it rose again in all groups. This finding seems in line with other studies, where the platelet count nadir was between 2 and 5 days after the procedure [1,6,7,8]. The reasons for such phenomena are so far not clearly elucidated, and hypotheses are based on studies conducted on surgical bioprostheses. Prior studies advocated, for example, the effect of nonphysiological shear forces, which can lead to the formation of von Willebrand factor multimers and platelet microaggregates [1,9,10]; smaller prosthesis diameter [1,11]; and the storage of prostheses in homocysteic acid [1,11,12]. It is important to point out that many patients affected by aortic valve disease are prone to developing coagulopathy, such as acquired von Willebrand syndrome (aVWS) [13]. Even in our study, a tendency of coagulopathy (as showed in the baseline higher activated partial thromboplastin time) compatible with aVWS was observed in the ST group. This observation suggests that preprocedure blood screening should be more thorough. Although prior studies highlighted that TAVI thrombocytopenia most often occurs in balloon-expanding valves [1], any association with the type of valve prosthesis has been found in our multivariable analysis. Indeed, the lower incidence of thrombocytopenia in our patients receiving a self-expandable prosthesis cannot lead to strong conclusions due to the smaller group size.

Although other studies have already investigated the incidence of thrombocytopenia after TAVI, the bias of perioperative fluid management has not previously been adequately addressed. If the hemodilution (e.g., perioperative fluid management) can cause a pseudothrombocytopenia, then the hemoconcentration (e.g., preoperative fasting) can occult baseline thrombocytopenia. As both hemodilution and hemoconcentration are difficult to measure, and their incidence could easily differ between centers, comparisons between studies in the literature are difficult. Recently, other colleagues managed this bias by adopting the corrected platelet count (CPC) as a reference in a study investigating thrombocytopenia following sutureless aortic valve replacement, and recommended the use of this value as a reference [3]. In our study, the application of this method revealed a significantly higher incidence of thrombocytopenia in comparison with the noncorrected count, despite a similar variation over time (with a nadir between days 2 and 3). This discrepancy between CPC and absolute values confirms the variation in hematocrit during the first postoperative days. To the best of our knowledge, ours is the first study applying this method to TAVI procedures, and we encourage further studies to always consider the use of CPC.

In the ST group, we could identify some factors associated with the onset of ST. Interestingly, alongside procedural variables, many differences were observed in baseline laboratory parameters. These findings could theoretically improve perioperative management, and, consequently, the outcomes of TAVI patients. For example, patients at risk of developing severe thrombocytopenia (i.e., baseline platelet count values near the lower limit of the normal range, undergoing a transapical approach or with a coagulopathy) should have ready-to-use concentrated platelets available.

It is worth highlighting the relationship between ST and life-threatening or major bleeding. Although the association between these two phenomena is clear, the relationship is far from obvious and requires further investigation. Two scenarios are possible: the patient has a bleeding event that results in subsequent thrombocytopenia, or the patient develops thrombocytopenia and, subsequently, a hemorrhagic event occurs. The low rate of this event makes it difficult to distinguish between the two scenarios. In our study, only 37 life-threatening bleeding events (of which 6 occurred in the ST group) were observed over a 9-year period. Further studies, possibly in a prospective fashion, should be designed ad hoc in order to discriminate between the two phenomena.

As the reported complications affect not only the patient’s morbidity but also the resource consumption, strategies for preventing thrombocytopenia and/or its consequences (prolonged staying in ICU and in hospital, higher incidence of bleeding) could determine economic benefits too.

### Limitations

Our study was a retrospective single-center analysis. Moreover, we focused only on intra-hospital outcomes. Some prosthesis groups were small in comparison with others, and, therefore, differences between models could not be assessed. As this was the first use of CPC in TAVI patients, further studies should confirm the discrepancy between corrected and noncorrected platelet count. To conclude, our study population consisted of elderly or high/intermediate-risk patients, making these results not directly applicable to a young or low-risk population.

## 5. Conclusions

Thrombocytopenia after TAVI is a phenomenon that significantly affects the onset of complications and in-hospital prognosis. Its incidence seems significantly higher than when observed using the noncorrected count. Our study lays the foundation for a better and deeper understanding of the possible mechanisms underlying this phenomenon. Patients at risk (e.g., with lower baseline count or undergoing TA-TAVI) of developing thrombocytopenia should be closely monitored to prevent complications from occurring. Future studies should investigate whether the correct management of modifiable variables would improve these outcomes.

## Figures and Tables

**Table 1:** Baseline characteristics of study population. Groups are defined according to the onset of postoperative thrombocytopenia. Values are expressed as mean [±standard deviation] or n (%). aPTT: activated partial thromboplastin time; AVR: aortic valve replacement; BMI: body mass index; CABG: coronary artery bypass grafting; COPD: chronic obstructive pulmonary disease; MV: mitral valve; PCI: percutaneous coronary intervention.

	Total Population (907)	No Thrombocytopenia (628; 69.24%)	Mild Thrombocytopenia (255; 28.11%)	Severe Thrombocytopenia (24; 2.65%)	*p*-Value
Age (years)	81.84 [±5.94]	81.71 [±5.78]	82.04 [±6.31]	82.94 [±5.99]	0.515
Women	436 (48.07%)	347 (55.25%)	111 (43.53%)	13 (54.17%)	**0.002**
BMI (kg/m^2^)	27.18 [±4.98]	27.23 [±5.03]	27.23 [±4.90]	25.42 [±4.07]	0.848
NYHA class	3.02 [±0.56]	3.02 [±0.58]	3.02 [±0.52]	3.02 [±0.51]	0.917
Critical preoperative state	34 (3.75%)	25 (3.98%)	6 (2.35%)	3 (12.50%)	0.167
Atrial fibrillation paroxysmal	90 (9.92%)	57 (9.08%)	32 (12.55%)	1 (4.17%)	0.098
Atrial fibrillation permanent	314 (34.62%)	212 (33.76%)	94 (36.86%)	8 (33.33%)	0.375
Prior pacemaker	87 (9.59%)	60 (9.55%)	26 (10.20%)	1 (4.17%)	0.7
Insulin dependent diabetes	92 (10.14%)	67 (10.67%)	24 (9.41%)	1 (4.17%)	0.649
Non-insulin-dependent diabetes	229 (25.25%)	160 (25.48%)	67 (26.27%)	2 (8.33%)	0.657
Chronic dialysis	26 (2.87%)	18 (2.87%)	8 (3.14%)	0	0.76
Extracardiac arteriopathy	259 (28.56%)	182 (28.98%)	69 (27.06%)	8 (33.33%)	0.533
Redo	60 (6.62%)	39 (6.21%)	19 (7.45%)	2 (8.33%)	0.527
Prior CABG	171 (18.85%)	123 (19.59%)	44 (17.25%)	4 (16.67%)	0.442
Prior AVR	52 (5.73%)	32 (5.10%)	18 (7.06%)	2 (8.33%)	0.283
Prior TAVI	2 (0.22%)	1 (0.16%)	1 (0.39%)	0	0.118
Prior valvuloplasty	14 (1.54%)	11 (1.75%)	3 (1.18%)	0	0.575
Prior MV replacement	10 (1.10%)	8 (1.27%)	2 (0.78%)	0	0.566
Prior MV repair	5 (0.55%)	4 (0.64%)	1 (0.39%)	0	0.686
COPD	191 (21.06%)	140 (22.29%)	44 (17.25%)	7 (29.17%)	0.079
Recent myocardial infarction	122 (13.45%)	91 (14.49%)	29 (11.37%)	2 (8.33%)	0.123
CCS grade 4	18 (1.98%)	15 (2.39%)	2 (0.78%)	1 (4.17%)	0.105
Liver disease	31 (3.42%)	23 (3.66%)	8 (3.14%)	0	0.771
Myelodysplastic disease	9 (0.99%)	6 (0.96%)	3 (1.18%)	0	0.727
Thyroid disease	272 (29.99%)	198 (31.53%)	68 (26.67%)	6 (25%)	0.172
Prior PCI	269 (29.66%)	193 (30.73%)	72 (28.24%)	4 (16.67%)	0.558
Euroscore II (%)	8.9 [±8.5]	9 [±8.5]	8.4 [±8.4]	10.5 [±7.4]	0.239
Echocardiogram					
Left ventricular ejection fraction (%)	53.09 [±12.84]	53.32 [±12.50]	52.73 [±13.73]	51.04 [±11.53]	0.595
Gradient max (mmHg)	74.46 [±24.04]	74.27 [±32.62]	74.72 [±32.98]	76.67 [±34.39]	0.847
Gradient mean (mmHg)	44.23 [±14.86]	43.93 [±18.04]	44.56 [±18.07]	48.5 [(±20.58]	0.684
Aortic valve area (mm^2^)	0.70 [±0.16]	0.70 [±0.25]	0.70 [±0.25]	0.68 [±0.21]	0.989
Laboratory parameter					
Hemoglobin (g/dL)	12.17 [±1.74]	11.98 [±1.76]	12.60 [±1.65]	12.66 [±1.24]	**<0.001**
Hematocrit (%)	36.78 [±4.77]	36.29 [±4.83]	37.85 [±4.52]	38.30 [(±3.12]	**<0.001**
Red blood cells (millions/µL)	4.11 [±0.57]	4.08 [±0.56]	4.17 [±0.57]	4.25 [(±0.54]	**0.035**
White blood cells (/µL)	7240 [±2250]	7450 [±2360]	6770 [±1890]	6670 [±1730]	**<0.001**
Platelet count (/µL)	224460 [±75840]	247220 [±73870]	172870 [±50860]	176880 [±54510]	**<0.001**
C reactive protein (mg/dl)	0.82 [(±1.70]	0.90 [±1.72]	0.64 [±1.66]	0.44 [(±0.59]	0.066
aPTT (s)	32.49 [(±9.63]	31.97 [±9.76]	33.65 [±11.43]	33.87 [±9.53]	**0.028**
Procedural parameter					
Transfemoral access	618 (68.1%)	446 (71%)	161 (63.1%)	11 (45.8%)	**0.004**
Prosthesis label size (mm)	25.4 [±2.3]	25.3 [±2.3]	25.7 [±2.4]	25.7 [±2.4]	0.547
Conversion to surgery	14 (1.5%)	7 (1.1%)	4 (1.5%)	3 (12.5%)	**<0.001**
Combined PCI	6 (0.6%)	2 (0.3%)	3 (1.1%)	1 (4.1%)	**0.036**

The significant *p*-values (<0.05) are marked in bold.

**Table 2:** Primary outcomes. Groups are defined according to the onset of postoperative thrombocytopenia. Values are expressed as mean [±standard deviation] or n (%).

	Total Population (=907)	No Thrombocytopenia (=628)	Mild Thrombocytopenia (=255)	Severe Thrombocytopenia (=24)	*p*-Value
ICU length of stay, days	2.79 [±4.7]	2.33 [±3.8]	3.73 [±6.2]	4.75 [±5.6]	**<0.001**
Hospital length of stay, days	11.56 [±8.7]	10.83 [±8.2]	13.17 [±9.5]	13.67 [±7.5]	**<0.001**
Life-threatening bleeding	37 (4%)	14 (2.2%)	17 (6.6%)	6 (25%)	**<0.001**
Major bleeding	119 (13.1%)	75 (11.9)	36 (14.1%)	8 (33.3%)	**0.008**
Ischemic stroke	13 (1.4%)	7 (1.1%)	6 (2.3%)	0 (-)	0.312
Hemorrhagic stroke	2 (0.2%)	2 (0.3%)	0 (-)	0 (-)	0.89
In-hospital mortality	45 (4.9%)	25 (3.9%)	16 (6.3%)	4 (16.7%)	**0.01**
30-day mortality	41 (4.5%)	25 (3.9%)	12 (4.7%)	4 (16.7%)	**0.015**

The significant *p*-values (<0.05) are marked in bold.

**Table 3:** Independent factors of severe thrombocytopenia at logistic regression analysis.

Univariate Model					Multivariate Model (Stepwise)			
	OR	95% CI		*p*-Value	OR	95% CI		*p*-Value
Baseline platelet count	0.986	0.977	0.994	<0.001	0.986	0.977	0.994	0.001
BMI	0.92	0.83	0.08	0.08	-	-	-	-
NIDDM	0.26	0.06	0.021	0.021	0.204	0.05	0.91	0.037
Combined PCI	19.15	1.68	0.001	0.001	-	-	-	-
Critical preoperative state	3.93	1.11	0.022	0.022	5.667	1.46	21.96	0.012
TA access ^a^	2.6	1.15	0.017	0.017	2.85	1.23	6.6	0.014

^a^ reference transfemoral. BMI = body mass index; NIDDM = noninsulin diabetes mellitus; PCI = percutaneous coronary interventions; TA = transapical.

## Data Availability

Data of the present study may be available after a reasonable request to the corresponding author if in line with our institutional regulations.

## Supplementary Materials

The following supporting information can be downloaded at: https://www.mdpi.com/article/10.3390/jcdd9110388/s1, Table S1: Time course of non-corrected platelet values according to onset of postoperatively thrombocytopenia; Table S2: Time course Mean, standard deviation, median, mininum und maximum of corrected platelet values (CPC) according to access (TF = transfemoral, TA = transapical); Table S3: Time course of Mean, standard deviation, median, mininum und maximum of corrected platelet values (CPC) according to postoperative daysstudy groups.
