# Supplementary material for: A New Definition of Thrombocytopenia Following Transcatheter Aortic Valve Implantation: Incidence, Outcome, and Predictors
Source: J Cardiovasc Dev Dis. 2022 Nov 9;9(11):388. doi: 10.3390/jcdd9110388 (PMC9697985; doi:10.3390/jcdd9110388)
Supplement: Supplementary file 1 [file jcdd-09-00388-s001.zip › jcdd-1963784-supplementary.pdf]

Table S1. Time course of non-corrected platelet values according to onset of postoperatively thrombocytopenia. Values are expressed as mean, standard deviation, median, mininum und maximum.

		Baseline	Day 0	Day 1	Day 2	Day 3	Day 4	Day 5	Discharge
Total	Mean	224,42	181,68	177,37	159,69	155,80	165,68	174,77	244,95
(n = 907)	St.Dev	75,81	75,91	63,73	60,90	64,54	61,75	62,58	97,58
	Median	212	165	166	149,5	146	155	166	231,5
	Min	101	62	42	42	47	49	38	34
	Max	854	615	711	645	842	433	462	886
MT	Mean	160,08	112,36	111,32	96,51	90,26	94,27	105,95	176,60
(n = 109)	St.Dev	52,37	33,93	29,84	24,41	23,81	24,97	33,23	82,54
	Median	144	103	106	93	87	89	97,5	157
	Min	101	62	53	62	53	52	54	56
	Max	378	198	221	200	193	198	208	481
ST	Mean	162,00	66,00	69,40	50,80	88,00	76,00	72,33	142,75
(n = 5)	St.Dev	46,11	18,00	19,50	11,58	35,97	19,97	39,83	117,89
	Median	161	66	71	47	95	81	63	114
	Min	106	66	42	42	47	49	38	34
	Max	230	66	95	71	129	93	116	309

Table S2. Time course of corrected platelet values (CPC) according to access (TF = transfemoral, TA = transapical). Values are expressed as mean, standard deviation, median, mininum und maximum.

		Day 0	Day 1	Day 2	Day 3	Day 4	Day 5	Discharge
TF	Mean	176.28	154.70	140.99	135.67	144.20	149.20	209.08
	Std.-Deviation	83.08	57.06	60.46	66.71	60.86	61.59	89.06
	Median	156.14	145.51	130.49	125.80	135.70	140.63	198.37
	Minimum	51.37	44.46	33.02	33.86	38.84	46.45	49.41
	Maximum	466.31	493.24	593.88	826.30	388.99	385.48	813.72
TA	Mean	153.39	155.70	136.11	133.59	139.71	153.37	240.17
	Std.-Deviation	68.66	66.57	59.14	64.53	60.65	64.25	101.95
	Median	137.34	141.39	124.74	119.66	131.47	143.44	227.91
	Minimum	40.15	28.41	28.14	41.55	34.81	25.80	26.09
	Maximum	475.75	566.79	357.32	406.12	410.63	411.94	726.25
	Mean	160.95	155.02	139.16	134.96	142.50	150.95	220.03
	Std.-Deviation	74.33	60.23	59.97	65.92	60.75	62.65	94.89
Total	Median	146.00	143.78	128.72	122.84	133.88	141.80	202.92
	Minimum	40.15	28.41	28.14	33.86	34.81	25.80	26.09
	Maximum	475.75	566.79	593.88	826.30	410.63	411.94	813.72

Table S3. Time course of corrected platelet values (CPC) according to study groups. Values are expressed as mean, standard deviation, median, mininum und maximum.

		Corrected_Platelet_Count_Day0	Corrected_Platelet_Count_Day1	Corrected_Platelet_Count_Day2	Corrected_Platelet_Count_Day3	Corrected_Platelet_Count_Day4	Corrected_Platelet_Count_Day5	Corrected_Platelet_Count_Day_Discharge
	Mean	192.31	176.45	164.41	163.80	172.85	181.65	245.80
No	Std.-Dev.	74.10	58.11	56.71	65.72	54.20	58.04	93.99
Thrombocytopenia	Median	169.93	163.59	148.82	150.05	160.02	171.29	228.34
	Minimum	102.54	102.06	100.26	100.47	100.30	100.52	103.15
	Maximum	475.75	566.79	593.88	826.30	410.63	411.94	813.72
	Mean	114.56	109.77	93.51	90.79	98.38	109.37	174.08
	Std.-Dev.	38.46	27.57	23.16	25.59	31.77	33.11	73.63

Mild Thrombocytopenia	Median	106.50	106.47	92.40	88.67	95.23	105.56	161.86
	Minimum	50.32	51.69	50.48	50.78	51.44	51.18	52.01
	Maximum	333.82	215.01	211.38	230.84	237.82	227.62	509.89
Severe Thrombocytopenia	Mean	82.15	80.29	57.16	60.76	57.01	63.87	138.00
	Std.-Dev.	47.12	37.43	28.26	32.01	14.57	22.03	98.38
	Median	58.26	68.75	51.22	50.99	56.24	57.06	99.18
	Minimum	40.15	28.41	28.14	33.86	34.81	25.80	26.09
	Maximum	155.54	173.35	170.50	183.48	87.33	104.25	325.99
Total	Mean	160.95	155.02	139.16	134.96	142.50	150.95	220.03
	Std.-Dev.	74.33	60.23	59.97	65.92	60.75	62.65	94.89
	Median	146.00	143.78	128.72	122.84	133.88	141.80	202.92
	Minimum	40.15	28.41	28.14	33.86	34.81	25.80	26.09
	Maximum	475.75	566.79	593.88	826.30	410.63	411.94	813.72

		Day 0	Day 1	Day 2	Day 3	Day 4	Day 5	Discharge
No Thrombocytopenia	Mean	192.31	176.45	164.41	163.80	172.85	181.65	245.80
	Std.-Dev.	74.10	58.11	56.71	65.72	54.20	58.04	93.99
	Median	169.93	163.59	148.82	150.05	160.02	171.29	228.34
	Minimum	102.54	102.06	100.26	100.47	100.30	100.52	103.15
	Maximum	475.75	566.79	593.88	826.30	410.63	411.94	813.72
Mild Thrombocytopenia	Mean	114.56	109.77	93.51	90.79	98.38	109.37	174.08
	Std.-Dev.	38.46	27.57	23.16	25.59	31.77	33.11	73.63
	Median	106.50	106.47	92.40	88.67	95.23	105.56	161.86
	Minimum	50.32	51.69	50.48	50.78	51.44	51.18	52.01
	Maximum	333.82	215.01	211.38	230.84	237.82	227.62	509.89
Severe Thrombocytopenia	Mean	82.15	80.29	57.16	60.76	57.01	63.87	138.00
	Std.-Dev.	47.12	37.43	28.26	32.01	14.57	22.03	98.38
	Median	58.26	68.75	51.22	50.99	56.24	57.06	99.18
	Minimum	40.15	28.41	28.14	33.86	34.81	25.80	26.09
	Maximum	155.54	173.35	170.50	183.48	87.33	104.25	325.99
